# Recalling fake news during real news corrections can impair or enhance memory updating: the role of recollection-based retrieval

**DOI:** 10.1186/s41235-022-00434-1

**Published:** 2022-09-16

**Authors:** Paige L. Kemp, Timothy R. Alexander, Christopher N. Wahlheim

**Affiliations:** Department of Psychology, University of North Carolina at Greensboro, 296 Eberhart Building, P. O. Box 26170, Greensboro, NC 27402-6170 USA

**Keywords:** Fake news, Facilitation, Interference, Recollection, Memory updating

## Abstract

Fake news can impair memory leading to societal controversies such as COVID-19 vaccine efficacy. The pernicious influence of fake news is clear when ineffective corrections leave memories outdated. A key theoretical issue is whether people should recall fake news while reading corrections with contradictory details. The familiarity backfire view proposes that recalling fake news increases its familiarity, leading to interference. However, the integrative encoding view proposes that recalling fake news promotes co-activation and binding of contradictory details, leading to facilitation. Two experiments examined if one theory better accounts for memory updating after participants recalled actual fake news details when reading headlines that corrected misinformation. In Phase 1, participants read real and fake news headlines of unclear veracity taken from various internet sources. In Phase 2, participants read real news headlines that reaffirmed real news and corrected fake news from Phase 1. When they detected that Phase 2 real news corrected fake news, they attempted to recall Phase 1 fake news. In Phase 3, participants first recalled real news details. When they remembered that those details were corrections from Phase 2, they attempted to recall fake news from Phase 1. Recalling fake news when noticing corrections in Phase 2 led to better memory for real news in Phase 3 when fake news was recalled again and worse memory for real news in Phase 3 when fake news was not recalled again. Both views explain part of the memory differences associated with recalling fake news during corrections, but only when considering whether people recollected that fake news had been corrected.

**Supplementary Information:**

The online version contains supplementary material available at 10.1186/s41235-022-00434-1.

## Significance statement

The proliferation of fake news in the media can create inaccurate memories that lead to negative effects on public beliefs, decision making, and health. Correcting fake news to mitigate its effects is sometimes effective, but it can also lead to interference in memory when corrections promote the retrieval of misinformation. Corrections facilitate the replacement of outdated misinformation with accurate details (i.e., memory updating), when people can recollect that those discrepancies had occurred. But corrections can also impede such updating when retrieving fake news details during corrections increases the feeling that misinformation details are familiar. The present study shows that these contradictory effects of corrections can be explained by a theory of memory updating proposing key roles for integrative encoding of real and fake news details, retrieval-enhanced familiarity of misinformation, and subsequent recollection of contradictory details and their relationship. The present findings suggest that successfully recalling fake news details when corrections are initially noticed can improve subsequent memory for real news. However, for this to be effective, recalled fake news must enable encoding processes that promote later recollection that fake news had been corrected. These findings support the recommendation that corrections should encourage comparisons of misinformation with accurate information, but only when the relationship between information is encoded well enough to be recollected.

## Introduction

The problem of “fake news” is a present dilemma. Fake news is false information presented via media outlets to persuade people that fictional ideas are factual. Exposure to fake news can create memory errors that serve as the basis for inaccurate beliefs. Reported associations between false beliefs and related behaviors suggest that such beliefs could have serious consequences. For example, beliefs based on memory for inaccurate claims about the COVID-19 virus are associated with reduced compliance with health guidelines and reluctance to vaccination (Roozenbeek et al., 2020). To mitigate these consequences, interventions will require clear targets for remediation. Since beliefs are partly based on memory accuracy, interventions will require identifying the mechanisms that allow people to distinguish between episodic memories of false and corrective information.

When corrections appear, the features shared with misinformation often cue retrieval of such information. This leads to repeated exposure that increases misinformation fluency and can further lead to impaired subsequent memory for corrections (Ecker et al., 2011; Schwarz et al., 2007). These observations have prompted the expert recommendation to avoid reminding people of misinformation during corrections (Lewandowsky et al., 2012) to avoid proactive interference. This view has recently been reversed (Lewandowsky et al., 2020) because research shows that repeating misinformation during corrections can enhance memory and belief accuracy by increasing conflict saliency and enabling integrative encoding (e.g., Ecker et al., 2017; Kendeou et al., 2014). In the present study, we examined how these opposing effects of misinformation retrieval during corrections on subsequent memory accuracy depend on whether participants use recollection-based retrieval. Here, we took a naturalistic approach by examining such mechanisms using stimulus materials comprising actual real and fake news headlines from internet sources.

The idea that retrieving misinformation during corrections can impair subsequent memory for those corrections has been accounted for by dual-process models of memory (e.g., Jacoby, 1991, 1999). Such models propose that retrieval practice increases the accessibility of misinformation by enhancing both recollection of context and acontextual familiarity (e.g., Bishara & Jacoby, 2008). When misinformation is updated by corrections, subsequent memory accuracy for corrections will partly depend on the balance of recollection and familiarity. Recollection of earlier-retrieved misinformation can oppose its familiarity and enable its rejection, whereas the absence of recollection leaves familiarity unopposed and allows misinformation to interfere with correct recall. Thus, retrieving misinformation during corrections should impair memory accuracy, but only when misinformation familiarity is unopposed by recollection-based retrieval (see, e.g., Butterfuss & Kendeou, 2020; Ecker et al., 2011). This interplay of retrieval processes is also considered to influence beliefs, such as when repeating information increases perceived truth (i.e., the illusory truth effect). This effect is considered to partly reflect familiarity misattributions (Begg et al., 1992; Schwarz et al., 2007), which is compatible with the view that familiarity can backfire when information sources are not recollected (also see, Skurnik et al., 2007). The illusory truth effect may also arise from repetitions increasing information coherence in semantic memory (Unkelbach & Rom, 2017; Unkelbach et al., 2019). Accordingly, the combination of increased coherence of misinformation from repetitions and decreased coherence of corrections due to its partial repetition of misinformation would lead misinformation to be relatively more fluent. This would impair later recall decisions when retrieved information about corrections is based on coherence and thus perceived truth.

Dual-process perspectives have also been invoked to account for the persistent effects of misinformation on indirect measures of memory and beliefs, such as reasoning, even after a correction (Johnson & Seifert, 1994; Wilkes & Leatherbarrow, 1988). The *Continued Influence Effect* (CIE) has been shown in paradigms wherein participants read a fictitious news report that contained a critical piece of information that was subsequently corrected or not corrected. The CIE is observed when corrections reduce, but do not eliminate misinformation reliance. One suggestion for why corrections are not completely effective is that they sometimes repeat misinformation, thereby increasing the potential for familiarity to exert an unwanted influence. However, the view that familiarity will backfire has recently been contested because the available literature shows that such effects are rare and depend on experimental design and stimulus characteristics (Swire-Thompson et al., 2020, 2022).

Evidence for the role of familiarity in the CIE has been recently shown in a study of the mechanisms of belief updating (Swire et al., 2017). Participants were presented with a series of true or false claims of unclear veracity and rated their beliefs in each. In the next phase, true claims were affirmed, and false claims were corrected. In a final phase, participants re-rated their belief in each statement either immediately or after a delay. Corrections reduced myth beliefs, but such reductions were smaller at longer delays. These findings were interpreted as showing that corrections of myth beliefs were sustained less effectively at longer delays because recollection was less available to oppose familiarity-based judgments. Although this implicates a role for familiarity in myth beliefs, this belief regression was not a true familiarity backfire effect because post-correction beliefs remained lower than pre-correction beliefs. Thus, familiarity backfire did not fully account for the CIE.

In contrast to the view that repeated exposure to misinformation impairs memory for and beliefs in corrections, others have suggested that it enhances updating by promoting awareness of information conflict, which provides the opportunity for integrative encoding. For example, in the traditional CIE paradigm where participants read narratives about events including incorrect details that are corrected in a subsequent narrative before an inferential reasoning test, corrections featuring an explicit misinformation reminder reduced the CIE better than corrections without a reminder (Ecker et al., 2017). The authors proposed that reminder benefits occurred because repeating misinformation fostered its co-activation with its correction. This presumably increased the salience of conflict between the competing information and supported integrative encoding and knowledge revision (Kendeou et al., 2014, 2019). These findings are consistent with work showing that detecting conflict can facilitate memory and belief updating (Putnam et al., 2014; Stadtler et al., 2013). These findings are also compatible with a retrieval account positing that conflict salience enhances the encoding of corrections, thus supporting their later recollection (Ecker et al., 2010; Seifert, 2002).

The findings above suggest that a comprehensive explanation of when misinformation retrieval during corrections will impair or improve memory requires considering the mechanisms proposed by familiarity backfire and integrative encoding accounts. The Memory-for-Change framework (MFC; Jacoby et al., 2015; Wahlheim & Jacoby, 2013) encompasses ideas from both accounts. The MFC framework proposes that stimulus features from a current event can cue retrieval of an earlier event, which enables detection of changed features between events. The co-activation of events then enables integrative encoding that supports subsequent source memory for event order when retrieval is recollection-based. However, the account also proposes that prior-event retrieval cued by current event features increases the familiarity of earlier events, which can lead to proactive inference effects when event changes are not subsequently recollected—a type of familiarity backfire. This mixture of effects has been shown consistently across paradigms using stimuli varying in naturalism (for a review, see Wahlheim et al., 2021). The generalizability of these findings suggests that whether retrieval of misinformation during corrections impairs or improves subsequent memory for corrections may similarly depend on whether recollection is used at retrieval.

The role of recollection-based retrieval in the effects of misinformation repetition on memory for corrections was recently shown using real-world news headlines (Wahlheim et al., 2020). Participants read real and fake news headlines of unclear veracity, and then read headlines that affirmed the factual headline or corrected the misinformation. Some of the corrections were immediately preceded by a fake news reminder, while others were not. Fake news reminders enhanced overall recall of details from corrective headlines, which supported an integrative encoding account and contradicted a familiarity backfire view. Importantly, conditional analyses of recall showed that these memory benefits reflected greater recollection-based retrieval. In contrast, familiarity backfired when fake news was not recollected in the form of lower correct recall of real news headlines and more false recall of fake news headlines. These findings suggested that fake news reminders can counteract the persistent effect of misinformation by cuing retrieval of earlier-fake news and promoting subsequent recollection of the conflict between fake and real news. However, the provision of fake news reminders precluded direct examination of the role of retrieving fake news details when detecting real news corrections. We addressed this limitation for theoretical interpretation here asking participants to overtly indicate when real news headlines corrected fake news headlines that they studied in an earlier phase and to recall the fake news headlines. Using this approach, this study examined associations between fake news retrieval when initial detecting real news corrections and subsequent memory for news details and their veracity.

Since the accessibility of earlier-studied misinformation should determine how often corrections are detected as such, manipulations of misinformation memorability should lead to differences in subsequent memory effects associated with retrieval practice of fake news when detecting corrections. One way to influence misinformation accessibility is to vary the congruence of participant and peer beliefs in the veracity of such information (Schwarz et al., 2016). From one perspective, when evaluating the veracity of information, people incorporate peer beliefs into their evaluations, especially when the information has ambiguous veracity (Gabbert et al., 2007) and social contacts endorse the belief (Galesic et al., 2021). In addition, when new information matches prior beliefs, encoding is more fluent (Schwarz et al., 2016), leading to stronger memory representations (Levine & Murphy, 1943), possibly by integrating information with schemas (Pratkanis, 1989). Accordingly, misinformation that both participants and peers believe would be more accessible, leading to better detection of contradictory details enabled by misinformation retrieval. This accessibility would also increase the risk that familiarity would backfire later when recollection is not engaged.

However, belief congruence does not always enhance memory, such as when contrasting it with belief-incongruent information leads the incongruent information to garner more attention (e.g., Maier & Richter, 2013). Related work has shown that incongruence between participant and peer beliefs may also increase misinformation accessibility when contradictions prompt critical processing of that information (Munnich et al., 2007) possibly in reaction to prediction errors piquing interest and attention (Vlasceanu et al., 2021). This would lead to stronger misinformation representations that better support memory for contradictory facts but also pose a greater risk for familiarity backfire when recollection fails. In our second experiment, we conducted an exploratory investigation on how the congruence between participant and peer beliefs affects misinformation encoding and subsequent memory for contradictory facts.

### The present study

The findings summarized above suggest that repeated exposure to fake news during corrections could impair or improve memory for corrections, depending on whether misinformation familiarity is opposed by recollection-based retrieval. However, no studies have directly examined how detection of real news that corrects earlier-studied fake news and retrieval of fake news during such detection leads to a mixture of improved and impaired memory for real news details. As mentioned earlier, our approach is inspired by work showing that memory, reasoning, and beliefs were improved when explicit reminders of misinformation were provided with corrections (Ecker et al., 2017; Wahlheim et al., 2020). However, those experiments did not directly assess the role of misinformation retrieval cued by corrections with shared features. The present study contributes to theory by illuminating how verified retrievals of fake news when detecting corrections are associated with subsequent memory updating. On the practical side, this experiment is more likely to resemble situations in everyday life in which people encounter corrections without the original fake headline or fact-checker tags.

This gap in the literature was addressed using a variant of the misinformation correction paradigm from Wahlheim et al. (2020). Participants first studied real and fake news headlines of unclear veracity. Next, they studied real news headlines that either reaffirmed the real news or corrected fake news. While studying these headlines, a prompt appeared asking participants to indicate instances when real news headlines corrected fake news and attempted to recall the fake news details when indicated as such. In the final phase, they attempted to recall real news details, indicated if those details had earlier corrected fake news, and if so, attempted to recall the fake news details.

Based on previous findings from similar paradigms in the episodic memory updating literature (for a review, see Wahlheim et al., 2021), we predicted that recalling fake news details when detecting real news corrections would facilitate memory for real news details when the fake news details could be subsequently recollected as having been corrected. In contrast, we predicted that recalling fake news details when detecting real news corrections would interfere with subsequent memory accuracy when the fake news details could not be subsequently recollected as having been corrected. This would occur because the familiarity and thus perceived truthfulness of misinformation would be unopposed (i.e., backfire), leading to poorer recall of correct details and more misinformation intrusions. These findings would be consistent with the MFC framework which subsumes familiarity-based and integrative encoding accounts of the continued influence of misinformation and posits a key moderating role for recollection-based retrieval. We also explored how congruence between peer and participant beliefs during initial fake news encoding interacted with this mixture of effects. We describe potential outcomes for this exploratory aim below when introducing the second experiment.

## Experiment 1

Experiment 1 characterized proactive effects of fake news exposure on memory for real news details when participants initially retrieved fake news details while studying corrections and subsequently recollected such details on a final memory test. This allowed us to determine when prompting participants to retrieve earlier-studied fake news details leads to the positive effects of memory updating as well as the negative effects of familiarity backfire. Importantly, recent research suggests that both mechanisms can contribute to overall memory performance, depending on whether retrieval of real news details is recollection-based (Wahlheim et al., 2020). Based on that research, we expected that recalling fake news details during corrections would lead to proactive facilitation in memory for real news when fake news is subsequently recollected as being corrected and proactive interference when fake news is not recollected. The balance of these effects, along with instances where fake news is not recalled, should therefore determine the direction and magnitude of proactive effects of memory for real news corrections in unconditioned summary scores.

## Method

The stimuli, data, and analysis scripts are available on the Open Science Framework (OSF) website: https://osf.io/xnvrj/. The research reported here was approved by the Institutional Review Board at The University of North Carolina at Greensboro (UNCG).

### Participants

The participants were 48 UNCG undergraduates (25 women, 23 men), ages 18–25 (*M* = 19.23, *SD* = 1.32). Our stopping rule was to test as many participants as possible in approximately one semester given lab resources with the final sample being a multiple of the three experimental formats. We were primarily interested in how proactive effects of memory differed based on whether fake new details that were recalled during corrections were subsequently recalled in Phase 3. However, we also conducted a sensitivity analysis after collecting the data to estimate the statistical power to detect the smallest effect size (odds ratio [*OR*]) of interest in Experiment 1. This corresponded to the difference in real news recall in the Control and Correction headline conditions (*OR* = 1.36). The analysis indicated that Experiment 1 had 71% power to detect an odds ratio of 1.36. A complete description of this analysis and its results can be found in Supplementary Information (henceforth Additional file 1: SI 1.1).

### Design

The experiment used a 3 (Headline Type: Repetition vs. Control vs. Correction) within-participants design.

### Materials

We selected from various internet sources 45 headline pairs that each included one fake news headline and its real news correction. The headlines were taken from sources such as news center websites (i.e., MSNBC, CBS News), well-known people (President Donald Trump, Bernie Sanders), government statistics websites, and folk myth websites. The fake news headlines were factual errors, and the real news corrections included factual details that contradicted the errors. All fake news headlines were originally portrayed as being true. Each headline pair corresponded to a unique topic. For example, the topic on the US president who took the most vacation days included the fake news headline, “President Obama took fewer vacation days than any other recent president,” and the real news headline, “President Clinton took fewer vacation days than any other recent president.” When preparing the experiment, we were able to find 41 topics related to US events described in various news sources. To increase the number of topics, we also included 4 urban myths (e.g., “Only older people need a flu vaccine.”). To foreshadow, when preparing Experiment 2, we found enough US events to replace the urban myths used here. Both experiments showed the same patterns related to the main hypotheses of this study, thus mitigating concerns about headline-specific effects.

For counterbalancing, we randomly divided the 45 headline pairs into three groups of 15. We rotated groups through Headline Type conditions, which created three experimental formats. Groups appeared equally often in each condition across participants. Note that Phase 1 included a mixture of fake and real news headlines depending on the condition, whereas Phase 2 included all real news headlines. Phase 1 included 30 headlines (15 fake news; 15 real news). Phase 2 included 45 headlines [15 real news from Phase 1 (Repetition); 15 real news that corrected fake news from Phase 1 (Correction); and 15 real news that appeared only in Phase 2 (Control)]. We included control headlines as a contrast condition against which to assess proactive effects of fake news exposure on memory for real news headlines.

Phase 3 included 45 questions corresponding to Phase 2 headlines that could be answered with either fake or real news details. For example, the question “Which recent president took the fewest vacation days?” could be completed with the fake news detail “*President Obama*” or the real news detail “*President Clinton*.”

### Procedure

**Fig. 1:**
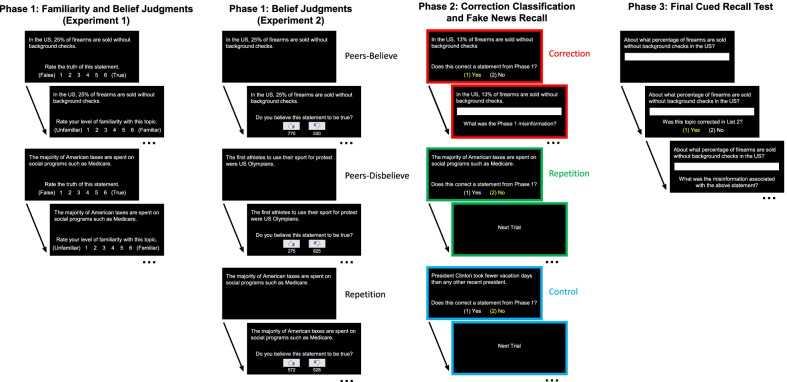
Schematic of the Procedure: Experiments 1 and 2. A schematic overview of the trial structures from the procedures in both experiments. The main difference between experiments was the trial structure in Phase 1: In Experiment 1, participants rated the familiarity and believability of headlines; In Experiment 2, participants rated the believability of each headline, which displayed the number of fictional peers who believed and disbelieved the headline. The majority of peers believed the headline in the *Peers-Believe* condition, and the minority of peers believed the headline in the *Peers-Disbelieve* condition. Phase 2 included *Correction* headlines that corrected fake news from Phase 1 (red borders), *Repetition* headlines that repeated real news from Phase 1 (green borders), and *Control* headlines that only appeared Phase 2 (blue borders). Note that all the trials in the *Peers-Believe* and *Peers-Disbelieve* conditions were later corrected, whereas that Phase 1 trials in the *Repetition* condition always included a negligible difference in peer beliefs. In both experiments, during Phase 2, participants indicated when they detected headlines that contradicted fake news, and if so, attempted to recall fake news from Phase 1. In the first slide of Phase 2 trials, the yellow highlights for the “Yes” and “No” judgments indicate the correct classification of each headline type upon which the second slide was contingent. During Phase 3, participants first recalled Phase 2 real news details, then indicated those for which fake news was corrected in Phase 2, and for those, attempted to recall the Phase 1 fake news

Participants completed the experiment individually in a quiet room with an experimenter present. Stimuli were presented electronically using E-Prime 2.0 software (Psychology Software Tools, 2012). Participants completed three phases in one session. Figure 1 displays a schematic of the procedure.

Prior to Phase 1, participants were told that their tasks would be to study individual headlines for an upcoming memory test and to rate the truthfulness and familiarity of each headline. In Phase 1, headlines appeared in a random order for 8000 ms each in white font against a black background. Two prompts then appeared below each headline sequentially for 5000 ms each. The first prompt asked participants to rate the truthfulness of the headline on a scale from 1 (False) to 6 (True). The second prompt asked participants to rate their familiarity with the headline on a scale from 1 (Unfamiliar) to 6 (Familiar). Participants were encouraged to use the full range of the scale and made their ratings by pressing a number on the keyboard. The prompt color changed from white to yellow after participants entered their ratings. Each slide was followed by a blank screen for 250 ms. The purpose of including these judgments was to obtain baseline assessments of beliefs and familiarity for all items and to give participants a task that would keep them actively engaged.

Prior to Phase 2, participants were told that some of the headlines they studied in Phase 1 were fake news and that corrections to those headlines would appear in Phase 2. They were also told that Phase 2 would include repetitions of real news headlines from Phase 1 and real news headlines that only appeared in Phase 2. Participants were told to study the headlines carefully for a later test and to indicate which headlines were corrections of Phase 1 fake news. During Phase 2, headlines appeared individually in a random order for 8000 ms each. Next, a prompt appeared below each headline asking participants to indicate if the headline corrected a headline that they studied in Phase 1. Participants made self-paced judgments, responding either Yes (1) or No (2) by pressing a number key. When they responded “Yes,” another prompt appeared above a text box wherein they typed only the fake news details from Phase 1 that were corrected in Phase 2 and pressed “Enter” to advance to the next headline. When they indicated “No,” the program advanced to the next headline. Note that the headlines stayed on the screen while participants made their responses. A mixed-effect model with Participants and Items as random intercept effects indicated that the estimated marginal mean of yes/no durations was significantly lower for Control (*M* = 1587 ms, 95% CI [1297, 1877 ms]) than Correction (*M* = 1969 ms, 95% CI [1679, 2259 ms]), *t*(2066) = 2.91, *p* = .01, but not Repetition (*M* = 1853, 95% CI [1564, 2143 ms]) headlines, *t*(2066) = 2.03, *p* = .11. Durations for Correction and Repetition headlines were not significantly different, *t*(2066) = 0.88, *p* = .65.

Prior to Phase 3, participants were told that their task would be to answer questions about the real news headlines they had just studied in Phase 2. During Phase 3, questions appeared individually in a random order above a text box until participants entered their response. After participants attempted to recall the real news details from Phase 2, the screen cleared, and a prompt asked whether the detail they typed had earlier corrected fake news from Phase 1. They responded by either Yes (1) or No (2) by pressing a number key. When they indicated “Yes,” a prompt asked participants to type in the fake news details from Phase 1 that were corrected in Phase 2. When they indicated “No,” the program advanced to the next item. Participants were encouraged to respond accurately to all items and were allowed to pass when they could not think of a response.

## Statistical methods

In both experiments, we performed all statistical tests using R software (R Core Team, 2021). To examine the effects of varying headline types, we fitted linear and logistic mixed-effects models using functions from the *lme4* package (Bates et al., 2015). Based on signal detection theory (SDT; Green & Swets, 1966) we also characterized participants’ detection of corrections during Phase 2 and subsequent memory that corrections had been detected during Phase 3 in terms of discrimination (*d’*) and response bias (*c*). We calculated these parameter estimates by computing hit and false alarm rates for each participant and using the *dprime* function from the psycho package (Makowski, 2021) to estimate the parameters. We performed Wald’s *χ*^2^ hypothesis tests using the *Anova* function of the *car* package (Fox & Weisberg, 2019) and post hoc comparisons using the Tukey method in the *emmeans* package (Lenth, 2021).

All models included Headline Type as a fixed effect as well as Participants and Items as random intercept effects to increase power (Miller et al., 2020). Given the self-paced feature of misinformation recall during Phase 2 study, we attempted to control for encoding time differences in the mixed-effects models of Phase 3 recall performance by including encoding time as a random effect. However, 11 out of 12 models did not converge. For the one model that did converge, the pattern of results was the same as when encoding time was not included. Consequently, encoding time is not included in the models reported below. The complete model specifications are available in the analysis scripts on the OSF: (https://osf.io/xnvrj/). The significance level was α = .05.

## Results

### Familiarity and belief ratings (Phase 1)

Participants’ familiarity with and beliefs in Phase 1 headlines were compared for real and fake news headlines by fitting separate models to each measure with Headline Type as a factor. The model for familiarity ratings indicated that participants were comparably familiar with real news (*M* = 2.38, 95% CI [2.06, 2.69]) and fake news (*M* = 2.45, 95% CI [2.14, 2.76]), *t*(1238) = 1.04, *p* = .30. In contrast, the model for belief ratings indicated that participants believed real news (*M* = 3.37, 95% CI 3.18, 3.57] more than fake news (*M* = 3.08, 95% CI [2.88, 3.27]), *t*(1241) = 3.74, *p* < .001. Note that the familiarity ratings were modest relative to the maximum possible rating, indicating that participants knew little about many of the headlines before entering the experiment.

### Cued recall performance (Phase 3)

We examined proactive effects of fake news exposure on memory for real news by examining correct cued recall of headline details from Phase 2. Cued recall responses were coded independently by two raters who were blind to the experimental conditions. Responses were considered correct when they included real news details from Phase 2 and intrusion errors when they included fake news details from Phase 1. These two response types are key measures of proactive effects of memory. Facilitation effects can be assessed in correct recall of recent information and interference effects can be assessed in both correct recall of recent information and errors originating from a non-recent source. Participants made other types of errors that were not of theoretical interest, so we do not report them here (see Additional file 1: SI 2 for a description of all response types and the scoring method used to classify responses).

#### Real news correct recall

**Fig. 2:**
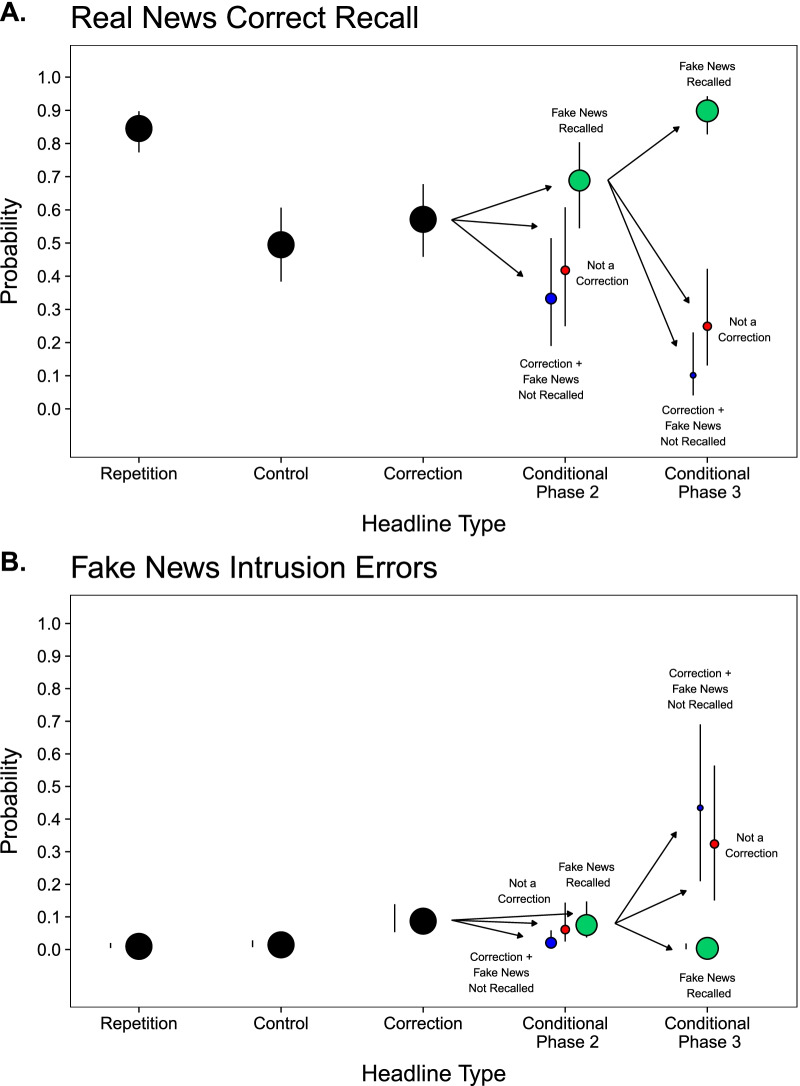
Recall of Real News and Intrusions of Fake News: Experiment 1. Probabilities of real news correct recall (Panel A) and fake news intrusion errors (Panel B) as a function of Headline Type in Experiment 1. Black points represent probabilities for all observations. Colored points represent probabilities conditioned on correction classification types in Phases 2 and 3. The cells represent corrections that were classified as such and for which fake news was recalled (green points), corrections that were classified as such and for which fake news was not recalled (blue points), and corrections that were not classified as such (red points). The size of each point indicates the relative proportion of observations in each cell. Error bars are 95% confidence intervals and are displayed adjacent to the points when the intervals lengths are shorter than point diameters

Figure 2A (black points) displays the overall probabilities of real news correct recall in Phase 3. A model including Headline Type as a factor indicated a significant effect, *χ*^2^(2) = 159.32, *p* < .001. Correct recall was significantly greater for Repetition than Control and Correction headlines, smallest *z* ratio = 10.09, *p* < .001, and for Correction than Control headlines, *z* ratio = 2.48, *p* = .04, indicating overall proactive facilitation in memory for real news that corrected fake news, and thus, no overall familiarity backfire effect.

#### Fake news intrusion errors

Figure 2B (black points) displays the overall probabilities of fake news intrusion errors in Phase 3. Note that intrusions for headlines in the Correction condition reflect episodic memory errors that occur when participants mistakenly report fake news details when asked to recall real news details. In contrast, intrusions for headlines in the Repetition and Control conditions are semantic memory errors that occur when participants spontaneously output fake news details that had not appeared in the experimental context. Therefore, the extent that there are more intrusion errors for Correction than Repetition and Control conditions indicates the contribution of proactive interference from fake news headlines that appeared in Phase 1. A model including Headline Type as a factor indicated a significant effect, *χ*^2^(2) = 96.54, *p* < .001. Intrusions were significantly higher for Correction headlines than Repetition and Control headlines, smallest *z* ratio = 7.50, *p* < .001, and not significantly different for Repetition and Control headlines, *z* ratio = 1.24, *p* = .43. These findings show that fake news exposure in Phase 1 led to proactive interference when participants attempted to recall of real news details from Phase 2.

#### Correction classifications and fake news recall (Phases 2 and 3)

To identify the role of detection of fake news corrections and recollecting that fake news had been corrected in cued recall accuracy in Phase 3, we first assessed participants’ awareness of and memory for such corrections in Phase 2 and Phase 3, respectively. We first computed the separate probabilities of participants indicating during Phase 2 that “yes” a headline was a correction and that during Phase 3 that “yes” the topic had earlier included a correction for all headline types. This provided a general indication of how well participants could discriminate Correction headlines from other headline types. Note that the extent to which participants incorrectly indicated that topics from the Repetition and Control conditions were associated with fake news corrections can be considered one index of response bias. Table 1 displays the probabilities of “yes” responses for each measure along with signal detection parameter estimates providing standardized estimates of discriminability (*d*’) and bias (*c*). In the signal detection analyses, the items in the Correction condition were treated as “old,” and the items in the Control condition were treated as “new.” We included Control headlines and excluded Repetition headlines, because Control headlines did not include features from Phase 1 and were thus more novel.

**Table 1:** Probabilities of correction classification and signal detection parameter estimates: Experiment 1

Phase	Classified as Correction (“Yes” Response)			Signal Detection Parameter Estimates	
	Repetition	Control	Correction	*d'*	*c*
Phase 2	.16 [.13, .18]	.03 [.02, .05]	.84 [.81, .87]	2.70 [2.48, 2.93]	.35 [.24, .45]
Phase 3	.15 [.12, .17]	.08 [.06, .10]	.72 [.68, .75]	2.05 [1.83, 2.28]	.41 [.30, .51]

95% confidence intervals are displayed in brackets

Awareness of and memory for corrections was first assessed by comparing the extent to which participants indicated that “yes” a headline topic had been (Phase 2) or was (Phase 3) a correction (Table 1, left panel) using a model including Headline Type and Phase (Phase 2 vs. Phase 3) as factors. The model indicated a significant effect of Headline Type, *χ*^2^(2) = 1236.54, *p* < .001, showing that probabilities were significantly higher for Correction than Repetition and Control headlines, smallest *z* ratio = 29.80, *p* < .001, and for Repetition than Control headlines, *z* ratio = 8.59, *p* < .001. There was also a significant effect of Phase, *χ*^2^(1) = 8.71, *p* < .01, that was qualified by a significant interaction, *χ*^2^(2) = 37.93, *p* < .001. The response probabilities for Correction headlines were significantly higher in Phase 2 than in Phase 3, *z* ratio = 5.71, *p* < .001, for Control headlines were significantly higher in Phase 3 than in Phase 2, *z* ratio = 3.70, *p* < .001, and for Repetition headlines were not significantly different between phases, *z* ratio = 0.62, *p* = .54. Together, these results show that participants’ ability to discriminate headlines that were corrected from the other headline types diminished from Phase 2 to Phase 3. This discrimination difference was verified by fitting separate models to estimates for each signal detection parameter (Table 2, right panel). The model for *d’* confirmed that discrimination was significantly higher during Phase 2 that in Phase 3, *t*(94) = 4.00, *p* < .001, whereas the model for *c* indicated no significant difference in response bias between phases, *t*(94) = 0.78, *p* = .44.

**Table 2:** Correction classification-type probabilities: Experiment 1

	Correction Classification Type		
Phase	Classified as Correction + Fake News Recalled	Classified as Correction + Fake News Not Recalled	Not Classified as Correction
Phase 2	.64 [.61, .68]	.20 [.17, .23]	.16 [.13, .19]
Phase 3	.50 [.47, .54]	.22 [.19, .25]	.28 [.25, .32]

95% confidence intervals are displayed in brackets. The classifications above all pertain to Correction headline types. “Classified as Correction + Fake News Recalled” were instances when participants indicated headlines that headline topics were associated with corrections and could recall the fake news details. “Classified as Correction + Fake News Not Recalled” were instances when participants indicated headlines that headline topics were associated with corrections but could not recall the fake news details. “Not Classified as Correction” were instances when participants indicated that headlines were not associated with corrections

**Fig. 3:**
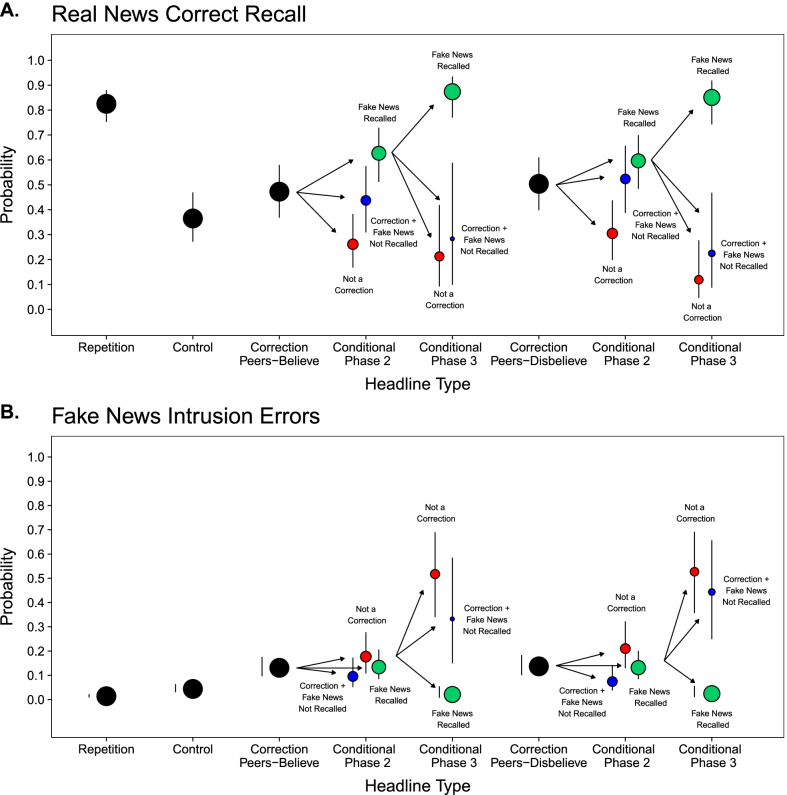
Recall of Real News and Intrusions of Fake News: Experiment 2. Probabilities of real news correct recall (Panel A) and fake news intrusion errors (Panel B) as a function of Headline Type in Experiment 2. Black points represent probabilities for all observations. Colored points represent probabilities conditioned on correction classification types in Phases 2 and 3. The cells represent corrections that were classified as such and for which fake news was recalled (green points), corrections that were classified as such and for which fake news was not recalled (blue points), and corrections that were not classified as such (red points). The size of each point indicates the relative proportion of observations in each cell. Error bars are 95% confidence intervals and are displayed adjacent to the points when the intervals lengths are shorter than point diameters

Next, we partitioned Correction headlines into three groups based on how participants classified them in Phase 2 and Phase 3 (Table 2). This allowed us to (1) assess participants’ recall of fake news headline details and (2) compute the proportions of observations comprising each cell in the conditional cued recall analyses (Fig. 3, colored points). *Classified as Correction* + *Fake News Recalled* refers to Correction headlines that were both classified as such (i.e., with a “yes” response) and for which fake news from Phase 1 was correctly recalled after the classification judgment. *Classified as Correction* + *Fake News Not Recalled* refers to Correction headlines that were classified as such and for which fake news from Phase 1 was *not* subsequently recalled. *Not Classified as Correction* refers to Correction headlines that were not classified as such (i.e., with a “no” response). Since the observations across these three cells were not independent, we only statistically compared the probabilities of fake news recall between the two phases (Table 2, left column) because those responses were of primary theoretical interest. The model indicated significantly higher fake new recall in Phase 2 than Phase 3, *χ*^2^(1) = 38.66, *p* < .001. We did not compare fake news recall in the Repetition and Control headline conditions because participants rarely reported headline details that had not appeared earlier in the experiment (*M* ≤ .03).

### Cued recall (in Phase 3) conditioned on correction classifications and fake news recall (from Phases 2 and 3)

Next, we examined how proactive effects of memory on cued recall accuracy in Phase 3 varied depending on whether corrections that were detected in Phase 2 and accompanied with the retrieval of fake news details were subsequently recalled as such in Phase 3. In the following analyses, we first examined associations between classifications made in Phase 2 and recall accuracy in Phase 3 with models including three levels of Classifications (Classified as Correction + Fake News Recalled, Classified as Correction + Fake News Not Recalled, and Not Classified as Correction) fitted to real news correct recall and fake news intrusions. We then examined how three levels of Classifications in Phase 3 for only when fake news was recalled in Phase 2 (Classified as Correction + Fake News Recalled) were associated with correct recall and intrusions using separate models for each. We conditioned the analyses on successful fake news recall during Phase 2 because those instances are of primary theoretical interest regarding whether retrieving earlier-studied headlines, would be associated with improved or impaired memory updating for real news headlines. Based on results from similar paradigms (e.g., Putnam et al., 2014; Wahlheim & Jacoby, 2013), we expected that recalling fake news details in Phase 2 would be associated with 1) facilitation in memory for real news details when fake news could subsequently be recollected and 2) interference when fake news could not be subsequently recollected.

#### Real news correct recall

Figure 3A (colored points) displays the conditional probabilities of real news correct recall in Phase 3. A model including Classification in Phase 2 (Classified as Correction + Fake News Recalled vs. Classified as Correction + Fake News Not Recalled vs. Not Classified as Correction) as a factor indicated a significant effect, *χ*^2^(2) = 28.82, *p* < .001, showing that correct recall was significantly higher for instances where corrections were classified as such and fake news was recalled (left green point) than when it was not recalled (left blue and red point), smallest *z* ratio = 3.59, *p* < .01. There was no significant difference in correct recall in the two cells for which fake news was not recalled in Phase 2, *z* ratio = 0.96, *p* = .60. Further, a model including the three types of Classifications in Phase 3 for when fake news was recalled in Phase 2 indicated a significant effect, *χ*^2^(2) = 90.77, *p* < .001, showing that recalling fake news was associated with enhanced recall of real news (right green point) compared to when fake news was not recalled in the two other cells (right blue point and red point), smallest* z* ratio = 7.56, *p* < .001. There was no significant difference in real news recall in the two cells for which fake news was not recalled in Phase 3, *z* ratio = 1.92, *p* = .13.

To assess proactive effects of memory, correct recall was compared between these Phase 3 conditional cells and Control headlines. Recalling fake news was associated with proactive facilitation, as correct recall was significantly higher than for Control headlines, *z* ratio = 9.21, *p* < .001. Collapsing across the other conditional cells for which fake news was not recalled, failing to recall fake news was associated with proactive interference, as correct recall was significantly lower than for Control headlines, *z* ratio = 5.52, *p* < .001. These results showed that overall recall of corrections reflected a mixture of facilitation and interference for which the balance depended on the extent to which participants engaged recollection-based retrieval.

#### Fake news intrusion errors

Figure 2B (colored points) displays conditional probabilities of fake news intrusion errors in Phase 3. The model fitted to intrusions conditionalized on the three Classifications in Phase 2 indicated a significant effect, *χ*^2^(2) = 9.03, *p* = .01, showing that intrusions when fake news was recalled (left green point) than when corrections were classified as such but fake news was not recalled (left blue point), *z* ratio = 3.00, *p* < .01. There was no significant difference in intrusions for when corrections were not classified as such in Phase 2 (left red point) compared to the other classifications (left green and blue points), largest *z* ratio = 2.25, *p* = .06.

The proportions of intrusions for only fake news recall in Phase 2 were then examined based on whether fake news was not recalled in Phase 3 (right blue point and red point). Intrusions for which fake news was recalled were not analyzed because those errors occurred when participants reported the fake news twice in succession, once as being the correct headline detail and once as being the fake news headline detail. These instances of guessing were rare (1%), but the cell appearing in Fig. 3B provides a complete picture of how the various response combinations comprised the overall intrusion probability for when fake news was recalled in Phase 2. The model for intrusions for when fake news details were recalled in Phase 2 conditionalized on whether they were not subsequently recalled (right red and blue points) indicated no significant difference, χ^2^(1) = 0.62, *p* = .43. These instances were then collapsed and compared with Control headlines to examine proactive effects of memory when fake news was not recalled. The model indicated that average intrusion rate of these cells was significantly higher than the baseline rate for Control headlines, *z* ratio = 10.79, *p* < .001. This is consistent with the results from correct recall showing that failing to recall fake news was associated proactive interference in memory for real news headlines, which is a form of familiarity backfire.

## Discussion

The results from Experiment 1 showed that correcting fake news increased correct recall for real news relative to when real news headlines appeared once in Phase 2 (i.e., proactive facilitation). These results are inconsistent with familiarity backfire accounts and are generally consistent with the integrative encoding account. However, conditional analyses revealed evidence for both familiarity backfire and enhanced updating that depended on whether participants could recall fake news and that it was corrected. These results extend those of Wahlheim et al. (2020) and are consistent with predictions from the MFC framework in showing a mixture of proactive facilitation when earlier-fake news details were retrieved during corrections and later recollected, and interference when earlier-fake news details were retrieved during corrections but not recollected. A similar finding was observed for fake news intrusion errors, showing that recalling earlier-fake news details during corrections but not recollecting corrections was associated with proactive interference. Collectively, these findings suggest that recollection-based retrieval opposed misinformation familiarity, which was increased when corrections were initially detected. The negative effects of recalling fake news details when detecting corrections shown in the absence of recalling those details as such in a subsequent phase join earlier results in showing that corrections can lead to familiarity backfire (Lewandowsky et al., 2012), but points out the key qualification that such backfire only occurred in specific instances. Thus, neither the familiarity backfire or integrative encoding account alone could explain the effects of recalling fake news details when detecting corrections on subsequent memory for real news headlines.

## Experiment 2

Experiment 1 characterized proactive effects of memory when fake news was retrieved during corrections and subsequently recollected. The patterns showing that successful fake news retrieval during corrections was associated with proactive facilitation when fake news was recollected and proactive interference when fake news was not recollected generalizes earlier findings (for a review, see Wahlheim et al., 2021). Experiment 2 examined the stability of those associations by testing whether they would replicate with an updated material set and slightly different experimental conditions. Experiment 2 also explored how a manipulation intended to affect encoding of fake news details during Phase 1 would subsequently affect the various memory measures in later phases. Since misconceptions about the veracity of everyday news content can be influenced by shared beliefs within social groups (Chan et al., 2017), we examined if varying peer agreement about the veracity of Phase 1 headlines would affect memory for headlines details and the ability to identify and remember fake news being corrected.

Participants were told that while reading each news headline in Phase 1, they should consider if it was true. Then, they were told that most members of a fictional peer group either believed or disbelieved the headline. This trial structure was intended to induce varying levels of congruence between peer and participant beliefs in a somewhat intermixed fashion. Belief congruence was assessed by requiring participants to rate their belief in each headline while a peer-belief message appeared. Based on work showing that belief-incongruent information captures attention (Munnich et al., 2007; Vlasceanu et al., 2021) and is more memorable than belief congruent information when the two are directed contrasted (e.g., Maier & Richter, 2013), which is inherent in the Experiment 2 procedure, we predicted that participants would attend more effectively to belief-incongruent headlines, resulting in better memory for fake news details shown on subsequent recall measures in Phases 2 and 3. If so, this should in turn improve memory for reals news, which was positively associated with correct recall of fake news in Experiment 1.

## Method

### Participants

The sample consisted of 76 UNCG undergraduates (53 women, 23 men) ages 18–27 (*M* = 19.05, *SD* = 1.66). As in Experiment 1, our stopping rule was to test as many participants as possible during one semester with the final sample size being a multiple of the total number of experimental formats, which was four (see below). A sensitivity analysis was conducted to estimate the observed power for detecting the smallest effect of interest, which was the difference in real news recall between the Control and Correction [Peers-Believe] condition. The present experiment had 99.50% power to detect an *OR* = 1.55 (see Additional file 1: SI 1.2).

### Design

The experiment used a 4 (Headline Type: Repetition vs. Control vs. Correction [Peers-Believe] vs. Correction [Peers-Disbelieve]) within-participants design.

### Materials

The material set included 60 headline pairs comprising one fake news headline and its correction. This set included the original 41 items corresponding to current events from Experiment 1 and 19 new items about other current events. Based on an item analysis of the Experiment 1 data, we also clarified the wording of three items from the Experiment 1 set. The materials are available on the OSF (https://osf.io/xnvrj/). For counterbalancing, headlines were rotated through conditions and appeared equally often in each condition across participants, as in Experiment 1. Phase 1 included 45 headlines (30 fake news; 15 real news). Phase 2 included 60 headlines [15 real news headlines repeated from Phase 1 (Repetition); 15 real news headlines that corrected Phase 1 fake news appearing earlier with an indicator showing that most peers *believed* the headline (Correction [Peers-Believe]); 15 real news headlines that corrected Phase 1 fake news appearing earlier with an indicator showing that most peers *disbelieved* the headline (Correction [Peers-Disbelieve]); and 15 real news headlines that only appeared in Phase 2 (Control)]. Phase 3 included 60 questions that queried the critical details of Phase 2 headlines.

### Procedure

Stimuli were presented electronically using E-Prime 3.0 software (Psychology Software Tools, 2016). The general procedure was like Experiment 1, but there were some key differences regarding Phase 1 (see Fig. 1). Prior to Phase 1, participants were told that their tasks would be to study headlines for an upcoming memory test and to silently consider whether they believed each headline was true. This aspect of the instructions was intended to induce mental models about headline veracity that could be affirmed or challenged by peer beliefs. Participants were told that data from previous participants’ judgments of truth would appear on a number counter that they would view while indicating their own belief. Participants were told the following cover story.

*The experiment in which you are about to participate is part of a multi-university study investigating the effects of misinformation and the influence of moderators such as social media. As a part of this collaboration, you will be contributing to a database of responses judging whether information is correct or not. When prompted, select whether you believe the statement to be true or false; you will see the live counter onscreen update to record your response. Thank you for your contribution.*

In Phase 1, each headline appeared in a random order for 8000 ms in white font against a black background. A prompt then appeared below the headline text for 5000 ms asking participants to indicate their belief in the headline. Participants made their responses by pressing on a “thumbs up” or a “thumbs down” emoticon, which represented “believe” and “do not believe” responses, respectively. A number counter appeared simultaneously below each button displaying the number of fictional peers who believed or disbelieved a headline. The integer shown on each counter was selected randomly for each trial from a range of percentages from a fictional sample of 1100 peers. In the Peers-Believe condition, the “believe” response counter showed an integer ranging from 65–75% of the total sample (e.g., 770), while the “do not believe” counter displayed an integer representing the remaining percentage (e.g., 330). In the Peers-Disbelieve condition, this proportion was reversed between counters.

We selected moderate values instead of more extreme values because more extreme values seemed less plausible from a participant’s perspective. However, we acknowledge that social influence can occur when extreme values are used (Kim, 2018; Vlasceanu & Coman, 2021). For Repetition headlines, the percentages ranged from 48–52% so that the presence of counters was not confounded with headline veracity. The prompt color changed from white to yellow and the counter updated when participants entered their ratings and remained on the screen for the entire 5000 ms trial duration. After Phase 1, participants completed a math distractor task for 10 min, indicating whether solutions to simple addition problems were even or odd numbers. This distractor task was included to lower the change detection rate from Experiment 1 (*M* = .87) to provide a more even distribution of items among cells for the recall analyses conditioned on Phase 2 correction detection responses. This modification was especially useful for examining differences in fake news intrusion errors (see below).

Phase 2 was the same as in Experiment 1. A mixed-effect model with Participants and Items as random intercept effects indicated that the estimated marginal mean of yes/no durations was significantly lower for Repetition (*M* = 1477 ms, 95% CI [1254, 1700 ms]) than Correction [Peers-Disbelieve] (*M* = 1770 ms, 95% CI [1547, 1993 ms]) headlines, *z* ratio = 2.79, *p* = .03. The estimate for Control headlines (*M* = 1741 ms, 95% CI [1518, 1964 ms]) was not significantly different than the estimates for Repetition, Correction [Peers-Believe] (*M* = 1620 ms, 95% CI [1397, 1843 ms]), Correction [Peers-Disbelieve] headlines, largest *z* ratio = 2.52, *p* = .06. Finally, there was no significant duration difference between the Correction [Peers-Believe] and Correction [Peers-Disbelieve] headlines, *z* ratio = 1.42, *p* = .49.

## Results

### Cued recall performance (Phase 3)

#### Real news correct recall

Figure 3A (black points) displays the overall probabilities of real news correct recall in Phase 3. A model including Headline Type as a factor indicated a significant effect, *χ*^2^(3) = 364.47, *p* < .001. Correct recall was significantly greater for Repetition than the other three headline types, smallest *z* ratio = 13.78, *p* < .001. Correct recall was also significantly higher for Correction [Peers-Believe] and Correction [Peers-Disbelieve] headlines than Control headlines, smallest *z* ratio = 4.35, *p* < .001. There was no significant difference between the Correction [Peers-Believe] and Correction [Peers-Disbelieve] headlines, *z* ratio = 1.25, *p* = .59. Replicating Experiment 1, these results indicate overall proactive facilitation in memory for real news that corrected fake news, and thus, there is no overall familiarity backfire effect.

#### Fake news intrusion errors

Figure 3B (black points) displays the overall probabilities of fake news intrusion errors in Phase 3. A model including Headline Type as a factor indicated a significant effect, *χ*^2^(3) = 204.22, *p* < .001. Intrusions were significantly higher for both types of Correction headlines than the other headline types, smallest *z* ratio = 8.21, *p* < .001. There were no significant differences between Correction [Peers-Believe] and Correction [Peers-Disbelieve] headlines, *z* ratio = 0.45, *p* = .97, but intrusions were significantly higher for Control headlines than Repetition headlines, *z* ratio = 5.40, *p* < .001. Replicating Experiment 1, these findings show that fake news exposure in Phase 1 led to proactive interference when participants attempted to recall of real news details from Phase 2.

### Correction classifications and fake news recall (Phases 2 and 3)

Following our approach in Experiment 1, we identified the role of detection of fake news corrections and recollecting that fake news had been corrected in cued recall accuracy by first assessing awareness of and memory for corrections in Phase 2 and in Phase 3 (Table 3). The extent to which participants indicated that “yes” a headline topic was or had been a correction (left panel) was compared between phases using a model including Headline Type and Phase as factors. The model indicated significant effects of Headline Type, *χ*^2^(3) = 2255.02, *p* < .001, showing that probabilities were significantly higher for Correction [Peers-Believe] and Correction [Peers-Disbelieve] than Repetition and Control headlines, smallest z ratio = 34.96, *p* < .001. There was no significant difference between the Correction [Peers-Believe] and Correction [Peers-Disbelieve] headlines, *z* ratio = 0.71, *p* = .89, or Repetition and Control headlines, *z* ratio = 0.83, *p* = .84. There was also a significant effect of Phase, *χ*^2^(1) = 99.55, *p* < .001, showing that probabilities were significantly higher in Phase 2 than in Phase 3. There was a significant interaction, *χ*^2^(3) = 48.80, *p* < .001, showing that the probabilities for Correction [Peers-Believe], Correction [Peers-Disbelieve], and Control headlines were significantly higher in Phase 2 than in Phase 3, smallest *z* ratio = 3.47, *p* < .001, and the probabilities for Repetition headlines were not significantly different between phases, *z* ratio = 1.87, *p* = .06. Collectively, these findings show that participants were less able to discriminate Correction headlines from other headline types in Phase 3 than in Phase 2.

**Table 3:** Probabilities of correction classification and signal detection parameter estimates: Experiment 2

	Classified as Correction (“Yes” Response)				Signal Detection Parameter Estimates			
					*d'*		*c*	
Phase	Repetition	Control	Correction [Peers-Believe]	Correction [Peers-Disbelieve]	Correction [Peers-Believe]	Correction [Peers-Disbelieve]	Correction [Peers-Believe]	Correction [Peers-Disbelieve]
Phase 2	.11 [.09, .13]	.14 [.12, .16]	.73 [71, .76]	.74 [.71, .76]	1.90 [1.68, 2.13]	1.89 [1.66, 2.11]	.26 [.15, .36]	.26 [.15, .37]
Phase 3	.13 [.11, .15]	.09 [.08, .11]	.58 [.55, .61]	.59 [.56, .62]	1.61 [1.39, 1.84]	1.63 [1.41, 1.86]	.58 [.47, .69]	.57 [.46, .68]

95% confidence intervals are displayed in brackets

To verify this claim, separate models were fitted to the estimates from each signal detection parameter (right panel). The model for *d’* indicated that discrimination was significantly higher during Phase 2 than in Phase 3, *F*(1, 300) = 5.74, *p* = .02. In addition, the model for *c* indicated that estimates were significantly higher in Phase 2 than in Phase 3, *F*(1, 300) = 33.36, *p* < .001, showing that participants became more conservative across phases. The less conservative responding in Phase 2, which did not replicate results from the first experiment, may have occurred because corrections comprised half of the items here and only a third of the items in the first experiment. No other effects were significant, *largest F*(1, 300) = 0.02, *p* = .88.

Also following our approach in Experiment 1, we next partitioned the classifications for Correction headlines in each phase into three types (Table 4) to assess the extent to which participants recalled fake news and to compute the proportions of observations comprising cells in the conditional analyses of cued recall (Fig. 3, colored points). A model including all headline conditions indicated no significant difference in fake news recall between the two Correction headline conditions, *z* ratio = 0.44, *p* = .97, and that fake news recall for those conditions was significantly higher in Phase 2 than Phase 3, smallest *z* ratio = 5.59, *p* < .001. Participants rarely reported details from the fake news headlines that had not appeared in the experiment for Repetition and Control headlines (*M* ≤ .02).

**Table 4:** Correction classification-type probabilities: Experiment 2

	Correction Classification Categories Probabilities					
	Classified as Correction + Fake News Recalled		Classified as Correction + Fake News Not Recalled		Not Classified as Correction	
Phase	Correction [Peers-Believe]	Correction [Peers-Disbelieve]	Correction [Peers-Believe]	Correction [Peers-Disbelieve]	Correction [Peers-Believe]	Correction [Peers-Disbelieve]
Phase 2	.50 [.48, .53]	.51 [.48, .54]	.24 [.20, .27]	.23 [.20, .27]	.26 [.22, .29]	.26 [.23, .30]
Phase 3	.41 [.38, .44]	.41 [.39, .44]	.18 [.15, .21]	.18 [.14, .21]	.42 [.38, .46]	.42 [.38, .46]

95% confidence intervals are displayed in brackets. The classifications above all pertain to Correction headline types. Classified as Correction + Fake News Recalled were instances when headlines were classified as corrections and participants could recall the fake news details. Classified as Correction + Fake News Not Recalled were instances when headlines were classified as corrections, but participants could not recall the fake news details. Not Classified as Correction were instances when headlines were not classified as corrections

### Cued recall (in Phase 3) conditioned on correction classifications and fake news recall (from Phases 2 and 3)

The role of detecting corrections and recalling fake news on the proactive effects of memory was examined using the same approach as in Experiment 1.

#### Real news correct recall

Figure 3A (colored point) displays the conditional probabilities of real news correct recall in Phase 3. A model including Classification in Phase 2 and Headline Type as factors indicated a significant effect of Classification, *χ*^2^(2) = 41.55, *p* < .001, no significant effect of Headline Type, *χ*^2^(1) = 0.25, *p* = .62, and no significant interaction, *χ*^2^(2) = 1.81, *p* = .40. Pairwise comparisons showed that correct recall was significantly higher when corrections were classified as such and fake news was recalled (left green point) than the other two classifications (left blue and red point), smallest *z* ratio = 2.52, *p* = .03. Correct recall was also significantly higher when corrections were classified as such (left blue point) than when they were not (left red point), *z* ratio = 3.59, *p* = .001.

Further, a model including the three types of Classifications in Phase 3 when fake news was recalled in Phase 2 and Headline Type as factors indicated a significant effect of Classification, *χ*^2^(2) = 80.45, *p* < .001, no significant effect of Headline Type, *χ*^2^(1) = 1.24, *p* = .27, and no significant interaction, *χ*^2^(2) = 0.48, *p* = .79. Pairwise comparisons showed that correct recall of real news was significantly higher when fake news was recalled (right green points) than when corrections were classified as such and fake news was not recalled (right blue points) and when corrections were not classified as such (right red points), smallest *z* ratio = 6.03, *p* < .001. There was no significant difference in correct recall in the two cells for which fake news was not recalled (right blue and red points) *z* ratio = 1.10, *p* = .52.

To examine the proactive effects of fake news exposure on subsequent memory for corrections, we first collapsed the Correction [Peers-Believe] and Correction [Peers-Disbelieve] conditions into a single Correction condition because there were no differences in conditional recall between these two conditions. We then compared conditional correct recall in the Correction headline conditions with the Control condition. A model including Headline Type as a factor with the three classification types for corrections in Phase 3 when fake news was recalled in Phase 2 and Control headlines as the levels indicated that recalling fake news was associated with proactive facilitation, as correct recall was significantly higher for those Correction than Control headlines, *t* ratio = 12.04, *p* < .001. Collapsing across the other conditional cells for which fake news was not recalled, failing to recall fake news was associated with proactive interference, as correct recall was significantly lower than for those Correction than Control headlines, *t* ratio = 3.88, *p* < .001. Replicating Experiment 1, these results show that overall recall of corrections reflected a mixture of facilitation and interference for which the balance depended on the extent to which participants engaged recollection-based retrieval. However, a novel finding here was that higher memory accuracy for real news when corrections were classified as such but no fake news details were retrieved relative to not classifying a correction.

#### Fake news intrusion errors

Figure 3B (colored points) displays conditional fake news intrusion errors in Phase 3. A model including Classification in Phase 2 and Headline Type as factors indicated a significant effect of Classification, *χ*^2^(2) = 11.65, *p* < .01, showing that there were fewer intrusion errors for when corrections were classified and fake news was not recalled (left blue points) than when corrections were not classified as such (left red points), *z* ratio = 3.43, *p* < .01. There was no significant difference in intrusions for when corrections were classified as such and fake news was recalled (left green points) compared to the other classifications (left blue and red points), largest *z* ratio = 1.93, *p* = .13. There was no significant effect of Headline Type, *χ*^2^(1) < 0.01, *p* = .97, and no significant interaction, *χ*^2^(2) = .92, *p* = .63.

The model for intrusions for when fake news details were recalled in Phase 2 conditioned on whether they were not subsequently recalled (right red and blue points) indicated no significant effect of Classification, *χ*^2^(2) = 2.20, *p* = .14, no significant effect of Headline Type, *χ*^2^(1) = 0.13, *p* = .72, and no significant interaction, *χ*^2^(1) = 0.20, *p* = .66. These instances were then collapsed and compared with Control headlines to examine proactive effects of memory when fake news was not recalled. The model indicated that average intrusion rate of these cells was significantly higher than the baseline intrusion rate for Control headlines, *t* ratio = 18.14, *p* < .001. As in Experiment 1, the overall patterns here are consistent with the results from correct recall showing that failing to subsequently recall fake news was associated proactive interference in memory for real news headlines, which is a form of familiarity backfire. However, the fewer intrusions associated with classifying a correction but not recalling fake news are a novel finding in this study.

### Peer beliefs (Phase 1) and memory for fake news corrections (Phases 2 and 3)

We also explored if the congruence between peer and participant beliefs influenced the encoding of fake news headlines in Phase 1 and its consequences for performance on memory measures in subsequent phases (for a complete description of these analyses, see Additional file 1: SI 3). A manipulation check revealed that peer-belief ratings influenced participant belief ratings in Phase 1: fake news headlines were rated as more believable when participants were told that most of their fictional peers also believed rather than disbelieved those headlines (Additional file 1: SI 3.1). However, contrary to our hypothesis that mismatching peer and participant beliefs would improve memory for fake and real news, there was no difference in fake news recall during Phase 2 and Phase 3 or in real news recall in Phase 3 depending on whether peer and participant beliefs matched or mismatched in Phase 1 (Additional file 1: SI 3.2).

## Discussion

Replicating Experiment 1, recall of real news was better when it corrected fake news than when it appeared only once during Phase 2. The overall enhancement in memory for real news resulting from it correcting fake news reflected a mixture of proactive facilitation and interference that depended on the extent to which fake news was retrieved during corrections and subsequently recollected. As in Experiment 1, recollecting earlier-retrieved fake news was associated with enhanced memory updating, whereas not recollecting earlier-retrieved fake news was associated with interference driven by familiarity backfire. This complex interplay could not be accounted for by the familiarity backfire or integrative encoding account alone. However, these findings are consistent with predictions from the MFC framework that subsumes the key mechanisms proposed by those accounts and proposes a moderating role for recollection-based retrieval. These findings build on Wahlheim et al. (2020) by showing that even without overt reminders of fake news headlines, retrieval of fake news leads to facilitation or interference in real news recall depending on if recollection-based retrieval is engaged on the final recall test. Finally, the manipulation of peer and participant beliefs showed no effects on misinformation accessibility, which was inconsistent with our hypothesis that belief-incongruent information would be better encoded and retrieved.

## General discussion

Research on misinformation corrections has shown that exposure to everyday misinformation has a continued influence on memories upon which beliefs are based (for a review, see Ecker et al., 2022). Given the current dilemma posed by the high prevalence of fake news in media outlets, determining ways to counteract its effects on memory accuracy is a priority. Despite the urgency of this issue, such effects are not yet well established. The primary goal of the present study was therefore to examine the role of recalling fake news details when initially detecting corrections and when attempting to subsequently recall the real news details that had corrected the fake news. The current experiments provided key data points for evaluating leading accounts of how fake news proactively affects memory for accurate information of the sort presented by news outlets. The present findings showed that retrieving fake news details during corrections can improve memory for real news headlines when fake news is later recollected and impair memory for real news headlines when fake news is not later recollected. The familiarity backfire and integrative encoding views could both partly account for different aspects of these findings, but the MFC framework better accounted for the complete pattern by subsuming key assumptions of those views and including a moderating role for recollection-based retrieval. In what follows, we discuss the implications of these findings for leading theoretical views and applications in everyday life.

### Familiarity backfire and recollecting detected corrections

Fake news corrections had once been discouraged because of concerns that they increase the fluency of misinformation by triggering its retrieval (Lewandowsky et al., 2012), but subsequent studies motivated a revision of that suggestion because corrections more often improve memory and belief accuracy (Lewandowsky et al., 2020). In fact, a recent review of the backfire effect literature (Swire-Thompson et al., 2020) concluded that belief-based familiarity backfire effects are not robust and are often an artifact of design characteristics that promote regression of beliefs to the mean, such as unreliable measures and pre–post-designs without a control group. Taken with the established finding that repeating headlines increases beliefs when corrections are absent (e.g., Fazio et al., 2019), Swire-Thompson et al. (2020) suggest pairing corrective information clearly with original misinformation, presumably to promote associative memory for details and their veracity and counteract erroneous fluency-based beliefs. However, this approach cannot always be achieved in the wild, especially when the rapid availability of media-based misinformation outpaces the release of fact-checked content, thus leaving people responsible for self-detecting corrections and deciding whether to engage retrieval mode to recall the original fake news details.

Although the present study did not directly examine how corrections influence beliefs, the findings did improve our understanding of when self-identified corrections improve or impair the memories upon which beliefs can be based. Consistent with prior findings (for a review, see Lewandowsky et al., 2020), the present results suggest that fake news corrections improved memory for updated headlines when misinformation had become associated with corrective information well enough to support recollection-based retrieval. These findings contribute to the backfire literature by highlighting the need to consider how identifying corrections by using their features to cue retrieval of misinformation can determine the extent to which backfire effects on memory occur *within* participants. This within-participant method of conditioning real news recall reveals how the influences of opposing processes are obscured when only analyses of aggregated data are considered. We therefore recommend that research aimed at identifying optimal correction formats leverage the conditional techniques used here and in related memory updating studies (e.g., Jacoby et al., 2015; Putnam et al., 2014). This approach could be useful in designing interventions that optimize participant strategies and correction details to promote misinformation retrieval and its associative encoding with corrective information.

### Integrative encoding of misinformation and corrections

The suggestion that associative encoding of misinformation and corrections can improve memory accuracy in the service of counteracting erroneous beliefs is central to integrative encoding accounts of memory and belief updating (e.g., Ecker et al., 2017; Kendeou et al., 2014; Wahlheim & Jacoby, 2013). According to those accounts, memory and belief updating are enabled when misinformation and correct information from two events becomes encoded together with a representation that identifies which details are true. Evidence for integrative encoding in memory paradigms can be inferred from response dependencies showing that updated information is more likely to be recalled accurately when original information is also recalled (for a review, see Wahlheim et al., 2021). The present finding showing that recall for real news was more accurate when earlier-retrieved fake news was subsequently recollected is compatible with an integrative encoding account of the proactive facilitation.

The present findings also replicate results showing similar facilitation in conditional recall of fake news headlines (Wahlheim et al., 2020) and build on those results by specifying the role of retrieving fake news details while encoding corrections. The present findings were also the first to show proactive facilitation in real news corrections in aggregate recall when participants were tasked with identifying corrections and retrieving fake news details on their own. This finding diverges from earlier results showing that corrections appearing without fake news details were remembered as well as real news that had not corrected fake news (Wahlheim et al., 2020). This discrepancy may have arisen because the correction detection procedure here that required generating prior-list fake news details encouraged participants to engage in study-phase retrievals more often, thus promoting integrative encoding for more headlines. This account is reminiscent of earlier findings showing that looking back to earlier information when encoding changes leads to proactive facilitation in recall of recently learned associations (Jacoby et al., 2015) via integrative encoding.

More broadly, the premise that integrative encoding facilitates updating also aligns with work in the educational literature that emphasizes the benefits of refutation texts on knowledge revision (Kendeou et al., 2014, 2019). These studies suggest that knowledge revision is successful when the refuted information is co-activated and integrated with the newly encoded correct information in memory. The present work broadens our knowledge of integrative encoding by illuminating its effects on memory updating in the context of fake news. Consistent with recent studies (Brashier et al., 2021; Grady et al., 2021), we used real-world news headlines that were corrected by fact-check verified headlines to enhance the applicability of our findings. Importantly, our analytic approach advances theoretical proposals in related work by identifying when integrative encoding was not effective and fake news produces interference by specifying a role for recollection-based retrieval.

### The role of recollection-based retrieval

The importance of recollection-based retrieval in overcoming interference is inherent in the dual-process account of the CIE (Jacoby, 1991, 1999; for a review, see Lewandowsky et al., 2012), which assumes that misinformation continues to exert its influence when automatic memory is unopposed by strategic recollection. Findings to corroborate this perspective come from studies showing how susceptibility to the CIE is heightened when recollection is less available, such as with older participants (Skurnik et al., 2005; Swire et al., 2017), longer retention intervals (Brashier et al., 2021; Walter & Tukachinsky, 2020), and when attention is divided (Ecker et al., 2011). However, a limitation of the dual-process account is that without modification it cannot account for findings showing that recollection of misinformation is associated with correct recall (Moore & Lampinen, 2016) or recent neuroimaging evidence implying that misinformation recollection drives the CIE and not misinformation familiarity (Brydges et al., 2020; Gordon et al., 2019). Together, these observations emphasize the need to consider a comprehensive account of misinformation correction effects that combines standard dual-process and integrative encoding perspectives.

### Cohort agreement

The present study also showed that the congruence between participant and peer beliefs about fake news accuracy in Phase 1 did not lead to differences in recall of fake news details in Phases 2 and 3 or in recall of real news details in Phase 3. This was somewhat surprising given that belief incongruence can induce skepticism, leading to a more analytic evaluation of headlines resulting from prediction errors that upregulate attention (Munnich et al., 2007; Vlasceanu et al., 2021). Although the reason for the absence of a belief congruence effect is unclear, one possibility is that belief congruence and incongruence both improved memory via different routes. For example, belief congruence could have enhanced encoding by improving encoding fluency (Schwarz et al., 2016), memory representations (Levine & Murphy, 1943), and integration with schemas (Pratkanis, 1989). Another possibility is that the values we selected for peer beliefs may have not been extreme enough to induce social influence (cf. Kim, 2018; Vlasceanu & Coman, 2021) that would stimulate elaborative processing when participants consider how their beliefs contradicted others. These possibilities could be examined by varying the extremity of peer beliefs and including a contrast condition for which peer beliefs are not disclosed to participants. Furthermore, the potential effects of belief congruence may have been limited because our materials included fairly partisan-neutral content. Since misinformation that aligns with political ideologies can be resistant to corrections (for a review, see Swire-Thompson et al., 2020), future research could examine how belief congruency interacts with memory while manipulating the alignment of misinformation content with participant partisanship.

### Inconsistent findings in the present experiments

While the present experiments converged in identifying the roles of integrative encoding and recollection-based retrieval in memory for corrections of fake news, there were a few unexpected differences in conditional recall performance. Unlike Experiment 1, in Experiment 2, cued recall accuracy in Phase 3 conditioned on classifications in Phase 2 showed that real news recall was higher when corrections were classified as such without fake news recall than those not classified as such and the inverse for fake news intrusions. Furthermore, both experiments showed no differences in real news recall or fake news intrusions when corrections for which fake news was recalled in Phase 2 were classified as such in Phase 3 without fake news recall and when they were not classified as corrections, which had not earlier been shown when using comparable materials (Wahlheim et al., 2020). Although there is no clear explanation, these discrepancies might reflect different bases for classifying headlines as having been corrected, such as partial recall of fake news details. However, a direct test of this supposition is required to determine its plausibility.

### On the relationship between memory and beliefs

While prior work has largely focused on the effects of misinformation exposure on beliefs (for a review, see Pennycook & Rand, 2021), a few studies have also examined proactive effects of misinformation on memory (Ecker et al., 2011; Wahlheim et al., 2020). We believe there is much to be gained from investigating the effects on both of these measures because memory is one primary basis for beliefs (e.g., Berinsky, 2017; Kowalski & Taylor, 2017). Evidence for this assumption comes from studies showing differences in belief regression that coincide with recollection differences. For example, myth beliefs following corrections regressed more to baseline beliefs at longer than shorter retention intervals and for older than younger adults (Swire et al., 2017). These results imply that when myth corrections were less well remembered, participants endorsed erroneous beliefs more. Taken with the present findings, these differences in belief regression inspire new avenues of inquiry about how beliefs vary based on the various retrieval combinations incorporating fake news recall measures across phases in paradigms such as those used here.

### Limitations

The present study had two primary limitations that were essential to note (though one could certainly identify others). First, the self-paced nature of the study phase could have contributed to memory differences for fake news corrections because participants spent slightly more time making judgments for those items in Experiment 1, and the headline remained on the screen until participants typed their recall response. This additional time could have been partly responsible for the proactive facilitation observed in overall real news recall. However, there was likely still a prominent role of integrative encoding in this recall advantage as an interference-based account would assume that more time spent encoding alternative associations when fake news was recalled would lead to subsequent proactive interference in overall recall. Future studies could control for encoding time differences by holding the duration constant across headline types and omitting Phase 1 recall following correction detection judgments. However, the average duration of correction detections was comparable between control and correction headlines in Experiment 2, providing evidence against a differential encoding account of proactive facilitation in overall recall.

Second, the stimulus materials and presentation format of corrections provided ecologically valid information content and a way to assess the downstream consequences of recalling fake news during corrections. Although consumers may read news headlines as short declarative sentences, as in the present study, headlines sometimes appear with images, and news is sometimes delivered in modalities with unfolding temporal structures, such as videos or podcasts. Additionally, it is not often that an outside source tells someone to read headlines and compare the content to existing memories for earlier-read headlines.

## Conclusion

To conclude, this study extends the line of research highlighting the importance of recollection-based retrieval following retrieval of fake news details during corrections for subsequent updating memory for real news details. It allowed us to test competing predictions from leading theoretical perspectives on memory and belief updating, namely the familiarity backfire and integrative encoding accounts. The present findings implicated roles for mechanisms from both accounts along with a critical moderating role for recollection-based retrievals. These findings suggest that successfully retrieving fake news details when reading real news headlines can promote the comparisons necessary to encode associations and support later recollection of news headlines and their veracity. Future interventions may be improved by considering how interactions between retrieval strategies engaged during encoding and the inclusion of overlapping features between real and fake news headlines can promote enduring memory representations upon which beliefs can be based.

## Supplementary Information

**Additional file 1:** Supplementary Information.

## Data Availability

The stimuli, data, and analysis scripts for Experiments 1 and 2 have been made publicly available via OSF and can be accessed at https://osf.io/xnvrj/.
