# Supplementary material for: Recalling fake news during real news corrections can impair or enhance memory updating: the role of recollection-based retrieval
Source: Cogn Res Princ Implic. 2022 Sep 16;7:85. doi: 10.1186/s41235-022-00434-1 (PMC9481799; doi:10.1186/s41235-022-00434-1)
Supplement: Supplementary file 1 — Additional file 1: Supplementary Information. [file 41235_2022_434_MOESM1_ESM.docx]

**Recalling Fake News During Real News Corrections Can Impair or Enhance Memory Updating: The Role of Recollection-Based Retrieval**

**Additional File 1: Supplementary Information**

Paige L. Kemp*, Timothy R. Alexander, and Christopher N. Wahlheim

**Corresponding author:* [*plkemp@uncg.edu*](mailto:plkemp@uncg.edu)

**1. Sensitivity Analyses**

**1.1. Experiment 1 Sensitivity Analyses**

We conducted a simulation-based sensitivity analysis using R software (R Core Team, 2020) based on the results from Experiment 1. The smallest effect size of interest in Experiment 1 corresponded to the difference in correct real news recall in Phase 3 between the Control and Correction headlines. This effect was of interest because it indexes the proactive effects of fake news exposure on memory for real news. To examine the sensitivity to detect this effect size, we first calculated the odds ratio of the pairwise difference between these conditions. To do this, we modeled the effects of experimental manipulations in Experiment 1 on real news recall in Phase 3 using logistic mixed effects models, fitted with the *glmer* function from the *lme4* package (Bates et al., 2015). We treated the predictor variable Headline Type as a fixed effect, and Subjects and Items as random intercept effects. We then conducted a significance test (z test) to derive the log odds ratio effect size for the difference between the Control and Correction headlines using the *dotest* function from the *simr* package (Green & MacLeod, 2016). We then converted the log odds ratio to an odds ratio using the *exp* function from *base R*. Finally, we used the *interpret_oddsratio* function from the *effectsize* package (Ben-Shachar et al., 2020) to interpret the size of the effect. The odd ratios for the advantage in real news recall for Correction over Control headlines was very small (*OR* = 1.36; Chen, 2010).

We then conducted the simulation-based sensitivity analysis using *simr* (Green & MacLeod, 2016) to examine the power to detect the odds ratio from the comparison described above. We fitted the real news recall data in Phase 3 with a logistic mixed-effects model that included a fixed effect of Correction headline type. A sensitivity analysis based on 1,000 simulations with alpha set at .05 revealed that with 48 participants, Experiment 1 had 70.70% [*95% CI* = 67.57, 73.31] power to detect a very small effect (*OR* = 1.36).

As a further step to determine what sample size would be required to detect a very small effect (*OR* = 1.36) with 80% power, we expanded the power stimulation curve to include a larger sample size. We increased the sample to 60 subjects using the *extend* function from the *simr* package. For the power curve analysis, we fitted the real news recall data in Phase 3 with a logistic mixed-effects model, including Correction headline type as the reference level. A power curve (see Figure S1) based on 1,000 simulations with 80% power and alpha set at .05 indicated that a sample size of 60 participants is required to detect an effect with a very small odds ratio (*OR* = 1.36). Thus, a sample size of this or larger would be appropriate for the second experiment.

**1.2. Experiment 2 Sensitivity Analyses**

Similar to Experiment 1, to examine the sensitivity to detect the smallest effect size of interest, we conducted a simulation-based sensitivity analysis in the same manner as for Experiment 1. In Experiment 2, the smallest effect of interest was the difference in correct real news recall in Phase 3 between the Control and Correction [Peers-Believe]) headlines. This effect was of interest because it assessed proactive effects of fake news exposure on memory for real news. Since was no difference in recall accuracy between the two Correction conditions, we selected the condition with the smallest difference from the Control condition. A sensitivity analysis based on 1,000 simulations with alpha set at .05 revealed that with a sample size of 76 participants, Experiment 2 had 99.50% [*95% CI* = 98.84, 99.84] power to detect a very small effect (*OR* = 1.55). A power curve based on 1,000 simulations with 80% power and an alpha set at .05 (Figure S2) indicated that a sample size of only 37 participants would have been sufficient to detect a very small effect (*OR* = 1.55). Experiment 2 was, therefore, well-powered to detect the smallest effect of interest.

**2. Cued Recall Scoring Method**

Phase 3 cued recall responses were classified into one of four types. *Real news recall* responses included correct details from Phase 2 headlines. *Fake news intrusion error* included details from false details from Phase 1 headlines. *Ambiguous* responses did not differentiate between facts and misinformation. *Other errors* included details that were inconsistent with either correct or fake news headlines as well as omissions. Two raters who were blind to experimental conditions independently coded responses into these four categories after being on a set of responses from a pilot study. The initial interrater agreement was almost perfect for the Phase 3 real news recall response coding in Experiment 1, *Cohen’s* $\kappa$ = .93, *p* < .001. Note that a single rater coded the fake news recall responses during Phases 2 and 3. The initial interrater agreement was almost perfect in Experiment 2, *Cohen’s* $\kappa$ = .92, *p* < .001, which included all responses in Phases 2 and 3. Discrepancies between raters were resolved through discussion.

**3. Exploratory Analyses of Peer Belief Manipulation in Phase 1**

**3.1. Belief Ratings for Phase 1 Headlines**

We first performed a manipulation check to determine whether participants on average believed more Phase 1 fake news headlines when most of their fictional peers also believed those headlines. For completeness, we also compared these with the rates of believing Phase 1 real news headlines. A model including Headline Type as a factor indicated a significant effect, χ^2^(2) = 129.00, *p* < .001, showing that participants indicated believing more fake news headlines in the Correction [Peers-Believe] (*M* = .55, *95% CI* = [.50, .60]) than Correction [Peers-Disbelieve] (*M* = .33, *95% CI* = [.29, .38]) condition, *z* ratio = 10.01, *p* < .001. Participants showed no significant difference in the proportion of headlines believed for real news in the Repetition condition (*M* = .55, *95% CI* = [.50, .60]) and fake news in the Correction [Peers-Believe] condition, *z* ratio = 0.16, *p* = .99, but they believed significantly more real news headlines in the Repetition condition than fake news headlines in the Correction [Peers-Disbelieve] condition, *z* ratio = 9.86, *p* < .001. These results confirmed that perceived peer belief influenced participants’ belief in fake news in the expected directions.

**3.2. Belief Congruence, Correction Classification, and Fake News Recall**

The cued recall results reported in the main manuscript showed enhanced memory updating associated with fake news recall in Phases 2 and 3. An exploratory aim of Experiment 2 was to determine if the measures of fake news recall in Phases 2 and 3 were sensitive to encoding differences in Phase 1 resulting from whether participant and peer beliefs were congruent. We tested the hypotheses that mismatches between participant and peer beliefs would stimulate more elaborative encoding and thus improve memory for fake news details in subsequent phases. Since fake news recall in Phases 2 and 3 was associated with facilitation in real news recall on the Phase 3 test, this also led to the hypothesis that real news recall would benefit from peer and participant belief mismatches. Performance on these memory measures was compared for *mismatched beliefs*, occurring when participants made a belief judgment that contradicted the fictional peer group, and *matched beliefs*, occurring when participants made a belief judgment that corresponded with the fictional peer group.

A Belief Congruence (Mismatched vs. Matched) × Phase (2 vs. 3) model fitted to fake news recall indicated a significant effect of Phase χ^2^(1) = 30.68, *p* < .001, showing that probabilities were significantly higher in Phase 2 (*M* = .51, *95% CI* = [.40, .63]) than in Phase 3 (*M* = .37, *95% CI* = [.27, .48]). Critically, there was no significant effect of Belief Congruence χ^2^(1) = 0.47, *p* = .49, showing that mismatches between participant and peer beliefs did not improve memory for fake news. There was no significant interaction, χ^2^(1) = 0.04, *p* = .85. Since belief congruence did not affect fake news recall in either phase, it was unlikely that it would affect real news recall in Phase 3. Indeed, a model fitted to real news recall in Phase 3 indicated no significant effect of Belief Congruence, χ^2^(1) = 1.18, *p* = .28.

**Figure S1**

*Experiment 1 Power Curve*

**
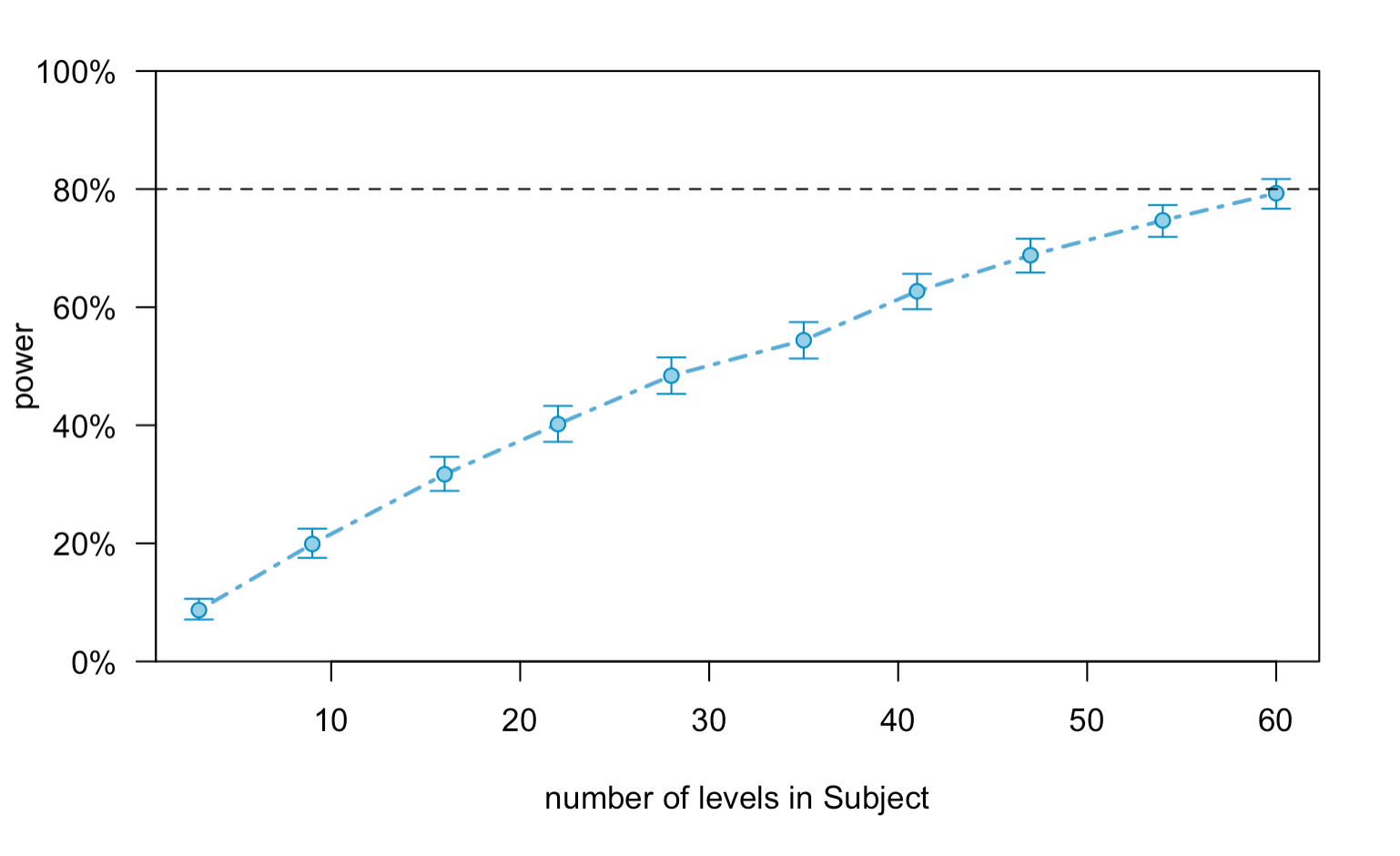
**

*Note.* Power curve to detect a very small effect (*OR* = 1.36) as a function of sample size (number of levels in Subject) using data from Experiment 1.

**Figure S2**

*Experiment 2 Power Curve*

**
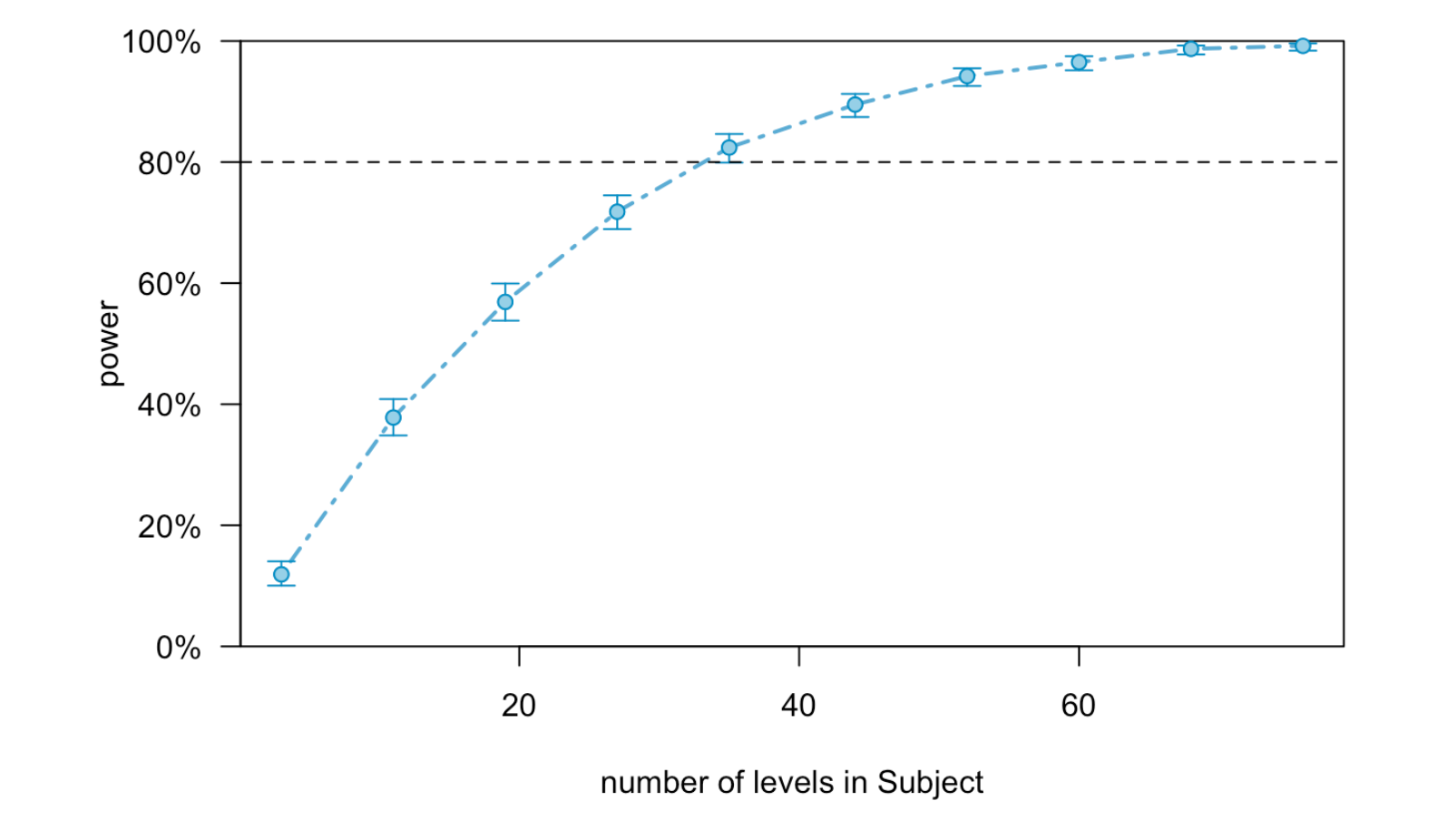
**

*Note.* Power curve to detect a small effect (*OR* = 1.55) as a function of sample size (number of levels in Subject) using data from Experiment 2.

**References**

Bates, D., Maechler, M., Bolker, B., & Walker, S. (2015). Fitting linear mixed-effects models using lme4. *Journal of Statistical Software, 67*(1), 1-48.

Ben-Shachar, M.S., Lüdecke D., & Makowski, D. (2020). “effectsize: Estimation of Effect Size Indices and Standardized Parameters.” *Journal of Open Source Software*,*5*(56), 2815. <https://doi.org/10.21105/joss.02815>.

Chen, H., Cohen, P., & Chen, S. (2010). How Big is a Big Odds Ratio? Interpreting the magnitudes of odds ratios in epidemiological studies. *Communications in Statistics - Simulation and Computation*, *39*(4), 860–864. <https://doi.org/10.1080/03610911003650383>

Green, P., & MacLeod, C. J. (2016). SIMR: an R package for power analysis of generalized linear mixed models by simulation. *Methods in Ecology and Evolution*, *7*(4), 493-498.

R Core Team (2020). R: A language and environment for statistical computing. R Foundation for Statistical Computing, Vienna, Austria. URL <http://www.R-project.org/>
